# From mechanistic modeling to AI-driven design: computational strategies for targeting the γ-secretase complex

**DOI:** 10.1093/bib/bbag231

**Published:** 2026-05-20

**Authors:** Sutapa Das, Shashank Rao Padubidri, Sreelakshmi K V, Koushik S Shetty, Rutuparna Jena, Himabindu K R, Budheswar Dehury, Arun Prasad Pandurangan

**Affiliations:** Department of Bioinformatics, Manipal School of Life Sciences, Manipal Academy of Higher Education, Manipal, Eshwar Nagar-Planetarium Complex, 576104, Karnataka, India; Department of Bioinformatics, Manipal School of Life Sciences, Manipal Academy of Higher Education, Manipal, Eshwar Nagar-Planetarium Complex, 576104, Karnataka, India; Department of Bioinformatics, Manipal School of Life Sciences, Manipal Academy of Higher Education, Manipal, Eshwar Nagar-Planetarium Complex, 576104, Karnataka, India; Department of Bioinformatics, Manipal School of Life Sciences, Manipal Academy of Higher Education, Manipal, Eshwar Nagar-Planetarium Complex, 576104, Karnataka, India; Department of Bioinformatics, Manipal School of Life Sciences, Manipal Academy of Higher Education, Manipal, Eshwar Nagar-Planetarium Complex, 576104, Karnataka, India; Department of Bioinformatics, Manipal School of Life Sciences, Manipal Academy of Higher Education, Manipal, Eshwar Nagar-Planetarium Complex, 576104, Karnataka, India; Department of Bioinformatics, Manipal School of Life Sciences, Manipal Academy of Higher Education, Manipal, Eshwar Nagar-Planetarium Complex, 576104, Karnataka, India; Department of Medicine, University of Cambridge, Cambridge Biomedical Campus, Papworth Road, Cambridge, CB2 0BB, United Kingdom

**Keywords:** γ-secretase, computational biology, molecular dynamics, AI-driven drug discovery, Alzheimer’s disease

## Abstract

Advancements in computational biology are transforming the study of complex membrane proteins and their therapeutic targeting. The γ-secretase complex, a quintessential intramembrane protease implicated in Alzheimer’s disease (AD) and more than 150 other substrates, provides a powerful exemplar to illustrate this transformative shift. Traditional γ-secretase inhibitors have been constrained by off-target toxicity, particularly through disruption of Notch signaling, underscoring the need for deeper mechanistic insights, now increasingly enabled by modern computational methodologies. We evaluate the computational strategies driving next-generation drug discovery of γ-secretase. Integrative modeling frameworks, informed by cryo-electron microscopy (cryo-EM) and biophysical data, have facilitated atomic-resolution reconstructions of γ-secretase dynamics and substrate recognition. All-atom molecular dynamics (MD) simulations, supported by enhanced sampling techniques such as umbrella sampling, steered MD, replica exchange, and Gaussian accelerated MD, have mapped conformational landscapes and elucidated molecular determinants of substrate selectivity. Structure–function mapping of familial AD mutations further demonstrates how computational modeling translates genetic variation into mechanistic understanding. Beyond structural modeling, the integration of artificial intelligence (AI) including deep generative models, machine learning-based activity prediction, and high-throughput virtual screening has created accelerated pipelines for discovering modulators predicted to reduce pathogenic amyloid beta (Aβ) production while preserving essential signaling pathways. These approaches demonstrate how computational methods increasingly serve as predictive and design-oriented engines in drug development. Using γ-secretase, this review highlights how state-of-the-art computational techniques, from integrative structural biology to AI-driven drug design, are reshaping the discovery of safer, more selective modulators with broader relevance across diseases requiring precise modulation of protein function.

## Introduction

The intramembrane protease complex known as γ-secretase plays a crucial role in cellular biology and neurodegenerative disease therapeutics. As a metalloprotein complex that regulates intramembrane proteolysis across a diverse range of type-I membrane substrates (Table 1), γ-secretase orchestrates a multitude of signaling events essential for development, tissue homeostasis, and neural function [1]. The enzyme complex is composed of four essential subunits: presenilin (PS1 or PS2), which provides the catalytic aspartyl active site; nicastrin (NCT); anterior pharynx-defective-1 (APH-1); and presenilin enhancer-2 (PEN-2). Together, they form a multimeric membrane-embedded holoenzyme that undergoes sequential assembly process, endoproteolysis of presenilin, and trafficking to endosomes, the plasma membrane, and other subcellular compartments [2]. The defining characteristic of γ-secretase biology is its remarkably extensive substrate repertoire; over 150 transmembrane proteins have been reported as substrates beyond the canonical amyloid precursor protein (APP) and the Notch receptor family [3]. This breadth underpins both its physiological importance and the therapeutic challenges it presents in disease contexts such as Alzheimer’s disease (AD).

**Table 1:** Summary on key computational studies on γ-secretase.

**Computational approach**	**System**	**Key findings**	**Biological insights**	**References**
**Enhanced sampling MD (LiGaMD3)**	γ-secretase–Aβ complex (Aβ40 and Aβ42 bound states)	Captured Aβ dissociation pathways and identified low-energy intermediate conformations during release	Revealed dynamic mechanism of substrate dissociation, critical for understanding processive proteolysis and AD pathogenesis	[23]
**Cryo-EM with molecular modeling and dynamics refinement**	Apo, substrate (Aβ_46_/APP-C99)-bound and inhibitor (MRK-560)-bound γ-secretase complexes	High-resolution cryo-EM structures captured substrate-free and intermediate-bound states; refinement via MD revealed substrate recognition and sequential ε → ζ → γ cleavage steps; isoform-selective inhibitor binding was elucidated (PS1 versus PS2).	Provided structural snapshots of processive proteolysis, clarified substrate engagement and catalytic transitions, and revealed isoform-selective inhibitor interactions guiding rational design of safer γ-secretase modulators.	[10, 24, 90]
**Gaussian accelerated MD (GaMD) and enhanced sampling simulations**	Full γ-secretase–APP-C99 complex and presenilin FAD mutants	Captured spontaneous activation, conformational transitions, and catalytic water positioning; revealed how FAD mutations shift the catalytic dyad equilibrium and disrupt APP alignment; identified hybrid β-sheet near scissile bond controlling stepwise cleavage.	Unveiled dynamic enzyme activation mechanism, mechanistic link between hybrid β-sheet stability and processivity, and mutation-induced disruption of ε-cleavage explaining altered Aβ profiles.	[15, 16, 25, 26]
**Free energy and Thermodynamic simulations (PMF, alchemical, umbrella sampling)**	APP-C99 transmembrane helix and presenilin active site mutants	Quantified cleavage energetics showing preference for Aβ_49_ versus Aβ_48_ pathways; calculated mutation-induced perturbations of catalytic geometry and energy landscapes.	Explained molecular basis of Aβ length heterogeneity and linked mutation energetics to altered catalytic efficiency useful for predictive mutational analyses and inhibitor screening.	[12, 27]
**pH-REMD and QM/MM multiscale simulations**	Catalytic dyad (Asp257/Asp385) in apo and substrate-bound γ-secretase	Demonstrated context-dependent dyad protonation; revealed protonation-controlled activation barriers and water-mediated proton transfers during hydrolysis.	Provided atomistic insight into acid–base catalysis and hydrolysis mechanism within the membrane informing design of mechanism-based inhibitors and accurate MD/QM/MM parameterization.	[28, 29]
**Structural modeling, docking and integrative analyses**	γ-Secretase–SERP1 (Aph-1A/NCT) complex; comparative dynamics and inhibitor modeling	Mapped interaction interfaces showing SERP1 enhances Aβ production while reducing Notch cleavage; integrated cryo-EM and MD findings explaining conformational plasticity and processive proteolysis.	Suggested selective modulation of subunits to alter APP versus Notch processing; integrated computational experimental insights supporting next-generation Alzheimer’s therapeutic design.	[4, 11]
**MD with biochemical/cellular validation**	FAD mutant presenilin complexes across multiple substrates	Showed that some mutations stall processing, forming toxic intermediate complexes independent of Aβ length changes.	Expanded view of Alzheimer’s pathology: stalled enzyme–substrate states can be pathogenic, suggesting therapeutic focus on restoring turnover rather than only modulating Aβ ratios.	[30]

Within the milieu of AD research, γ-secretase has emerged as a compelling therapeutic target largely through the lens of the amyloid hypothesis, and the accumulation of amyloid-β (Aβ) peptides, particularly the Aβ_42_ isoform, initiates and drives the neurodegenerative cascade. The sequential proteolysis of APP by β-secretase followed by γ-secretase constitutes the final step in the generation of Aβ fragments. Thus, inhibiting or modulating γ-secretase appears to be a logical route to reduce pathogenic Aβ formation [4, 5]. However, early efforts focused on pan inhibitors of γ-secretase inhibitors (GSIs) ran headlong into a paradox; although Aβ production could be lowered, the blockade of γ-secretase activity also disrupted the cleavage of other essential substrates, most notably Notch, with consequent toxicity, altered cell-fate signaling, immunosuppression, gastrointestinal epithelial abnormalities, and worsened cognition [6, 7]. In mouse and human trials, nonselective GSIs generate unacceptable adverse events and, in some instances, cognitive decline, forcing a reevaluation of the therapeutic strategy [8]. The realization that γ-secretase inhibition must spare vital substrate pathways has given rise to the concept of a Notch-sparing modulator, a compound that shifts the cleavage profile of γ-secretase to reduce pathogenic Aβ without disabling physiological substrate processing.

In response to these therapeutic constraints, the class of γ-secretase modulators (GSMs) has emerged as a more nuanced alternative to complete inhibition. Rather than abolishing enzyme activity, GSMs act allosterically to adjust the cleavage site preferences of γ-secretase, essentially biasing the enzyme toward the production of shorter, less amyloidogenic Aβ species such as Aβ_38_ or Aβ_39_ while preserving the core processing of other substrates such as Notch. Preclinical work has validated that GSMs reduce Aβ_42_/Aβ_40_ ratios and mitigate plaque burden in transgenic models without overt Notch-related toxicity [9]. More recently, second-generation GSMs have been optimized for greater potency, brain penetrance, and favorable pharmacokinetics. Nevertheless, despite these promising developments, the mechanistic basis of γ-secretase substrate selectivity, the molecular effects of pathogenic familial AD (FAD) mutations, and the design of next-generation modulators remain constrained by our current structural, dynamic, and computational understanding of the complex as a membrane-embedded proteolytic machine.

The rationale for advancing computational biology as a key enabler in this domain is compelling. The membrane-embedded, multi-subunit architecture of γ-secretase, the intricate nature of its conformational states, its sequential, processive catalytic mechanism that trims Aβ and its promiscuous substrate repertoire collectively present significant challenges for conventional structural and functional analysis. In this context, computational strategies encompassing integrative modeling [combining cryo-electron microscopy (cryo-EM), nuclear magnetic resonance (NMR), mass spectrometry, and Förster resonance energy transfer (FRET)/electron paramagnetic resonance (EPR) constraints], all-atom molecular dynamics (MD) simulations, including enhanced-sampling frameworks such as umbrella sampling, steered MD, replica exchange, Gaussian accelerated MD (GaMD), and AI-driven small-molecule design (machine learning for activity prediction, deep generative models, and virtual screening at the ultra large scale), are rapidly revolutionizing the discovery pipeline. These methodologies not only facilitate the description but also enable prediction and design of modulator molecules that selectively inhibit γ-secretase cleavage away from pathogenic Aβ while preserving Notch signaling, which represents a central therapeutic objective.

Specifically, the structural elucidation of γ-secretase has had a transformative impact. Recent cryo-EM maps of human γ-secretase in both apo and substrate-bound forms have revealed the active site architecture embedded within the lipid bilayer, the trajectories of substrate transmembrane helices entering the enzyme, and the role of the nicastrin ectodomain in substrate recognition [10]. Integrative modeling has bridged lower-resolution cryo-EM data with cross-linking mass spectrometry and EPR/NMR constraints to reconstruct flexible substrate enzyme assemblies and the initial docking states of both APP-C99 and Notch [11]. These structural frameworks serve as the foundation for the mechanistic dissection of substrate recruitment, binding, and processive proteolysis by γ-secretase.

Complementing structural snapshots, molecular dynamics simulations have elucidated key functional motions of γ-secretase in its membrane environment. These simulations have unveiled gating mechanisms, water penetration pathways, the influence of lipid composition in enzyme dynamics, and substantial conformational transformations that underpin the catalytic cycle [12]. Enhanced sampling MD approaches provide access to the free-energy landscapes and conformational transitions underlying γ-secretase catalysis and processive substrate trimming. GaMD has been used to capture the structural rearrangements that enable tripeptide trimming of Aβ_49_ during sequential cleavage, including the positioning of catalytic aspartates and water required for peptide bond hydrolysis [13]. Complementary steered MD simulations have further been applied to probe substrate unfolding and translocation through the PS1 catalytic core, offering mechanistic insight into differences between amyloidogenic processing pathways [14]. In addition, enhanced sampling studies demonstrate that substrate-enzyme hybrid β-sheet stability governs cleavage-compatible active-site geometry and water accessibility, thereby controlling efficient proteolysis [15]. Notably, computational mutagenesis and enhanced-sampling molecular dynamics studies of FAD-associated PS1 mutations have demonstrated that these variants shift the conformational equilibrium of γ-secretase away from catalytically competent states, leading to substrate misalignment and disruption of active-site geometry. Mechanistic insights from GaMD simulations further reveal that several PS1 FAD mutants impair APP positioning and alter the spacing between catalytic aspartates, thereby reducing ε-cleavage efficiency [16]. Such mutation-induced equilibrium shifts provide a mechanistic basis for the elevated Aβ_42_/Aβ_40_ ratio characteristic of pathogenic PS1 variants [17], consistent with broader computational analyses linking mutation-specific biochemical changes to clinical severity [18]. Parallel to structural and dynamic modeling, the contemporary chemical-biology and drug-design toolkit has embraced artificial intelligence (AI). Machine learning models trained on known GSM and GSI chemical series are used to predict activity and selectivity features. High-throughput virtual screening (HTVS) of ultra large chemical libraries via structural ensembles of γ-secretase enables the identification of candidate modulators that engage allosteric and substrate-binding pockets [19, 20]. Deep generative models such as generative adversarial networks (GANs), variational autoencoders (VAEs), and reinforcement-learning frameworks now enable *de novo* design of modulators optimized for multi objective profiles, such as high potency, low Notch interference, drug likeness, and brain penetration [21]. This synergy of integrative structural biology, advanced simulation, and AI-driven ligand design offers a paradigm shift from descriptive modeling to predictive, generative, design-oriented discovery.

While previous reviews have often discussed γ-secretase structural biology, substrate processing, or modulator development in isolation [4, 22], the rapid convergence of cryo-EM-guided hybrid modeling, enhanced-sampling molecular simulations, computational mutagenesis, and AI-driven ligand design now enables a more unified perspective. Bringing these advances together provides an opportunity to connect conformational transitions and processive proteolysis with mutation-driven mechanistic shifts and emerging strategies for the rational discovery of next-generation Notch-sparing γ-secretase modulators. Such an integrated computational viewpoint underscores the growing role of *in silico* approaches in guiding therapeutic innovation for complex intramembrane proteases.

In this review, we position γ-secretase not only as a central therapeutic target in AD pathogenesis, but also as a compelling illustration of how computational biology is reshaping the drug-discovery landscape for complex intramembrane proteases. We critically examine how integrative structural modeling, dynamic simulation and AI-driven small-molecule design converge to decode the substrate-selective proteolytic machinery, translate familial mutation biology into mechanistic insight, and drive the rational design of Notch-sparing modulators with therapeutic potential. The broader implications extend beyond AD, and the frameworks developed here can be applied to other intramembrane proteases relevant to developmental, oncologic, and metabolic diseases where selective modulation of proteolysis is vital.

## Deciphering the proteolytic machine: integrative structural biology of γ-secretase

### From low-resolution maps to atomic reconstruction: the Cryo-EM revolution

The γ-secretase complex epitomizes a sophisticated multicomponent proteolytic apparatus that orchestrates the regulated intramembrane proteolysis of many substrates, including APP and Notch, which are central to AD pathogenesis and cell fate determination, respectively [23]. For over a decade, the absence of high-resolution structural information on γ-secretase hindered progress toward rational drug design, given its integral membrane localization and multi-subunit assembly. Early electron microscopy and X-ray studies furnished only low-resolution outlines, insufficient to elucidate architectural details or mechanistic subtleties [24].

**Figure 1:**
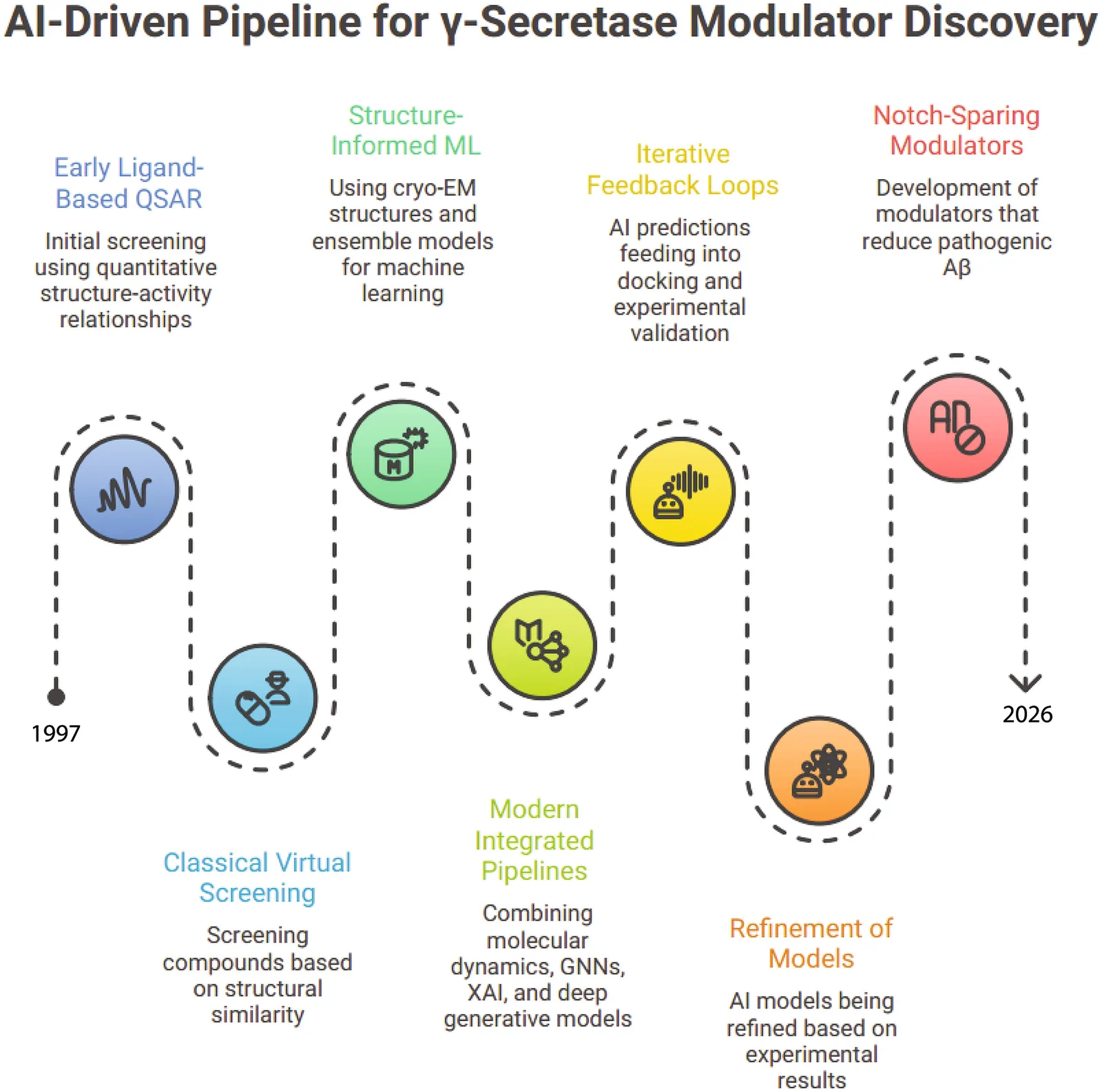
**Chronological evolution of AI-driven γ-secretase modulator discovery.** The schematic illustrates the chronological evolution of computational strategies, from early ligand-based QSAR and classical virtual screening to structure-informed machine learning enabled by cryo-EM data. This is followed by modern integrated pipelines combining molecular dynamics, graph neural networks, and generative AI within iterative feedback loops.

The advent of high-resolution cryo-EM has since revolutionized membrane protein structural biology, providing transformative insights into the architecture and function of γ-secretase. A modern iterative computational pipeline for γ-secretase drug discovery illustrates advances beyond classical virtual screening based on structural similarity (Fig. 1). Starting with cryo-EM-informed ligand-QSAR relationships, the workflow builds initial screening models, which feed into machine learning and docking predictions through iterative feedback loops; this generates refined AI models incorporating experimental validation. Concurrently, integrative pipelines combine classical X-ray similarity screening with modern approaches like MD, graph neural networks (GNNs), and deep learning to predict Notch-sparing modulators that selectively reduce pathogenic Aβ production. The process culminates in experimental refinement and validation of AI-derived leads, enabling a closed-loop discovery cycle for safer Alzheimer’s therapeutics.

Key structures, such as the 3.4 Å cryo-EM map of human γ-secretase in the apo state and the subsequent atomic models of substrate-bound forms, have revealed an intricate horseshoe-like arrangement of the four constituent subunits: presenilin (the catalytic core), Nicastrin, APH-1, and PEN-2 nicastrin ectodomain capping the entry vestibule, potentially regulating substrate access through steric gating. Substrate-bound cryo-EM structures featuring engineered APP and Notch peptides have illuminated the trajectory of substrate helices and resolved putative substrate-binding cavities, clarifying the molecular underpinnings of selectivity and catalysis [25]. These groundbreaking structures provide a mechanistic template for inhibitor and modulator development, directly visualizing drug-binding pockets, the spatial constraints imposed by the transmembrane region, and the conformational changes associated with catalysis. Combined with mutational mapping and pharmacophore modeling, such data establishes the structural foundation for next-generation therapeutics targeting the γ-secretase complex.

### Integrative modeling: bridging resolution gaps with computational hybrid methods

Despite the recent advancements in cryo-EM, several challenges persist. Flexible regions, transient conformational states, and the native lipid environment often remain elusive for static, ensemble-average methods. To address these limitations, integrative modeling frameworks employ hybrid approaches that combine cryo-EM density maps with complementary biophysical data, including NMR, FRET, EPR, and cross-linking mass spectrometry (XL-MS) [26, 27]. These hybrid strategies effectively reconcile sparse, often low-resolution or ambiguous experimental data by guiding computational modeling toward conformations consistent with the comprehensive array of available information. For example, NMR restraints define interatomic distances and validate secondary structures, while FRET and EPR report on subunit proximities and dynamic ranges between domains under near-physiological conditions. XL-MS data, by capturing distance constraints between lysine residues, further delineates topological relationships across subunits, which can otherwise be occluded in cryo-EM maps owing to motion or local disorder.

Within γ-secretase, such hybrid protocols have successfully resolved the flexible C- and N-termini of presenilin and the elusive interface between nicastrin and substrate peptides, domains typically obscured owing to inherent molecular plasticity. In particular, flexible substrates such as the APP transmembrane domain can be modeled into rigid scaffolds via integrative docking, conformational sampling, and energetics-based scoring, restoring a more complete, functionally relevant structural view [12]. The synergy between diverse datasets not only enhances spatial resolution but also enables the modeling of transient intermediate states crucial for understanding substrate recognition and entry step processes likely to differentiate APP from Notch binding and cleavage outcomes. The integration of bioinformatics and evolutionary constraints provides further value. Coevolving residue pairs identified by multiple sequence alignments often map to functionally relevant contact points, which are refined via computational modeling against experimental frameworks [28]. This combined computational and experimental paradigm is vital for developing atomistic models that can be reliably used for mechanistic interrogation and structure-guided small-molecule design.

### Visualizing the substrate recruitment and binding process

A major unresolved question has been elucidating how γ-secretase distinguishes among its diverse substrates while maintaining specificity despite its catalytic promiscuity or how the enzyme accommodates a broad substrate repertoire while retaining regulated and substrate-specific proteolysis. Consequently, recent advancements in cryo-EM and integrative approaches have begun to reveal the molecular choreography underlying substrate recognition and recruitment and when interpreted alongside integrative modeling and molecular dynamics analyses, these findings indicate that this apparent promiscuity arises not from a static active site but from a highly dynamic, conformationally adaptive proteolytic machinery. In particular, coordinated motions within presenilin transmembrane helices and regulatory elements of the complex enable γ-secretase to sample multiple substrate-compatible states [29]. Substrate recruitment is initially regulated by the nicastrin ectodomain, which acts as a dynamic gate rather than a rigid filter, undergoing transient opening and closing motions that permit access only to substrates with compatible N-terminal conformations [30]. Following entry, cryo-EM structures reveal that substrate transmembrane helices, including APP and Notch, engage a lateral groove within presenilin. Molecular dynamics simulations further show that this groove is conformationally plastic, with fluctuations in helix spacing, tilt, and side-chain rearrangements allowing accommodation of substrates with distinct sequence, length, and helical geometry [31].

Importantly, enhanced conformational sampling and computational mutagenesis demonstrate that γ-secretase does not rely on a single binding mode; instead, dynamic substrate-binding pockets transiently form and dissolve, enabling differential stabilization of APP versus Notch substrates. Disease-associated mutations that perturb the flexibility or electrostatic profile of these regions shift the conformational equilibrium toward nonproductive or misaligned binding states, thereby altering cleavage efficiency and promoting pathogenic amyloidogenic outcomes. Furthermore, distinct binding sites or subsites within presenilin have been identified that accommodate peptide backbones and side chains based on sequence, length, and helical geometry. Mutational analyses of these substrate-binding grooves revealed how substrate affinity and cleavage patterns determine selectivity. In this context, disease-associated mutations, especially those altering the groove’s shape or charge profile, can profoundly alter substrate positioning, resulting in diminished cleavage efficiency and pathogenic amyloidogenic outcomes.

Together, these findings indicate that γ-secretase substrate promiscuity is an emergent property of its dynamic architecture rather than a lack of specificity. Overall, visualization of the substrate recruitment and proteolytic engagement through a combination of cryo-EM, chemical cross-linking, and advanced modeling reveals the selective machinery of γ-secretase and its potential for therapeutic targeting. Elucidating these determinants not only enhances our mechanistic understanding but also holds translational promise: by identifying regulatory gating points unique to pathological substrates, computationally driven design of Notch-sparing modulators becomes feasible, mitigating the dose-limiting toxicities of broad-spectrum inhibitors.

## Simulating dynamics and mechanisms: molecular dynamics to rescue

### Capturing the functional motions of a membrane-embedded complex

While static structures have illuminated the architectural blueprint of γ-secretase, its catalytic proficiency and substrate selectivity are fundamentally governed by dynamic motions spanning sub-nanosecond side chain rotations to millisecond-scale domain rearrangements within its native membrane environment. All-atom MD simulations provide a unique window into these temporally and spatially resolved processes, yielding insights inaccessible to ensemble cryo-EM snapshots [32].

The embedding of a complete, atomistically detailed γ-secretase complex into a realistic lipid bilayer surrounded by explicit water and ions has revealed the spectrum of functionally relevant conformational changes underlying catalysis. These approaches capture transient opening and closing of substrate entry sites, gating dynamics of the nicastrin lid, vibrational flexing of presenilin helices, and the rearrangement of catalytic aspartyl motifs crucial for peptide bond scission. Water dynamics, simulated within the intramembrane cleft, demonstrate how transient hydration shells facilitate proton transfer and lower the activation barrier of hydrolysis, a key adaptation of aspartyl proteases situated within hydrophobic environments [33].

Moreover, MD simulations elucidate the conformational plasticity that underpins γ-secretase’s substrate promiscuity: subtle variations in helix bending, side chain rotamers, and domain swiveling are captured, revealing how the enzyme accommodates diverse substrates while retaining a degree of selectivity [34]. By mapping the frequency and amplitude of these motions, researchers have identified “hotspot” regions involved in allosteric regulation that potentially serve as druggable sites for Notch-sparing modulation [35].

### Enhanced sampling techniques for elucidating energetic landscapes

The conformational landscape traversed by γ-secretase is rugged, and many transitions, such as substrate insertion, repositioning for cleavage, and product egress, occur on timescales exceeding conventional MD capabilities. Consequently, enhanced sampling methods, including umbrella sampling, steered MD, replica exchange MD (REMD), and GaMD, have become indispensable in exploring these rare yet critical events.

Umbrella sampling, by restraining the system along predefined coordinates, enables the calculation of the free energy profiles governing substrate processing, most notably, the sequential generation of Aβ peptides from APP (Aβ_48_ → Aβ_42_ → Aβ_40_ → Aβ_38_) [36, 37]. These quantitative profiles reveal the energetic cost of each cleavage event, delineating the points at which familial Alzheimer’s mutations or small molecules might shift the balance toward pathogenic Aβ_42_ enrichment. Steered MD, by applying an external force, actively shuttles the substrate along the hypothesized processing pathways, demonstrating the mechanical feasibility and intermediate states accompanying substrate translocation and β-peptide cleavage [38].

REMD and GaMD overcome kinetic bottlenecks by enhancing configurational sampling, enabling the observation of large-scale conformational transitions such as nicastrin lid opening, presenilin helix rearrangements, or the formation/disassembly of substrate-binding pockets. These rare events, which are elusive on microsecond timescales, are functionally critical for discriminating between productive and nonproductive substrate engagement as well as for mapping allosteric sites that may be targeted for modulator design [39]. Enhanced sampling, in conjunction with free energy calculations, consequently elucidates the energetic topography underlying substrate selection, catalysis, and inhibition, thereby informing the mechanistic impact of both mutations and ligands.

### Mapping the molecular impact of pathogenic mutations

Computational mechanistic modeling has achieved a defining milestone by establishing the link between genotypic variation, such as FAD mutations in presenilin-1, and phenotypic outcomes at the molecular and cellular levels [40]. *In silico* mutagenesis and trajectory-based analysis facilitate the simulation of the structural and energetic consequences of specific amino acid substitutions, tracing how these changes perturb conformational equilibria, substrate positioning, or catalytic geometry and processive cleavage pathways.

**Figure 2:**
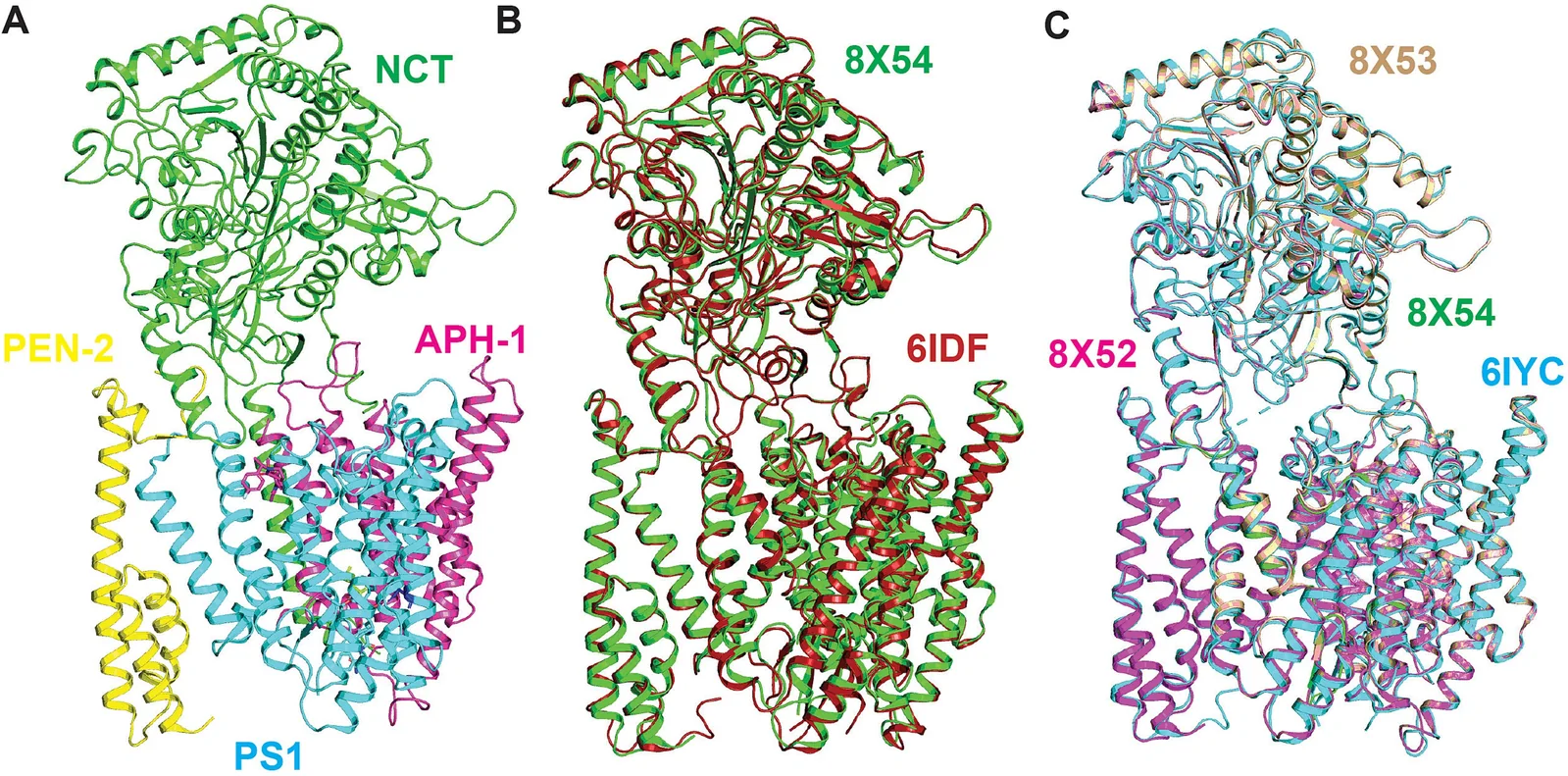
**Overlay of experimental Cryo-EM structures of γ-secretase bound with hybrid models of substrates of γ-secretase**. (**A**) Composite structural ensemble of the human γ-secretase complex showing its four core components: Nicastrin (NCT), APH1, Presenilin-1 (PS1), and PEN2. The catalytic dyad residues of PS1 (Asp257 and Asp385) are highlighted to indicate the location and geometry of the active site within the membrane-embedded cavity. (**B**) Overlay of γ-secretase bound to the Notch100 substrate (PDB: 6IDF; substrate shown in firebrick) and the APP-C99 fragment (PDB: 8X54; substrate shown in dark green). In this panel, only the substrate transmembrane regions are shown to emphasize differences in substrate trajectory and positioning within presenilin; the protein complex is displayed as a semitransparent surface for clarity. (**C**) Comparative overlay of APP-derived substrates within the γ-secretase active site, including C83 (PDB: 6IYC; substrate shown in olive green), Aβ49 (PDB: 8X52; magenta), Aβ46 (PDB: 8X53; teal), and C99 (PDB: 8X54; dark green).

Across multiple independent simulation studies, pathogenic FAD mutations are shown to induce subtle yet functionally significant effects. Simulations of pathogenic mutations frequently reveal subtle yet significant alterations; destabilizing helix packing, shifting the orientation of substrate peptides, or altering the hydration pattern within the catalytic core, including misalignment of APP-derived substrates within the active site, all of which influence the energy barriers for various cleavage steps. Notably, many FAD mutations skew the cleavage propensity of γ-secretase toward longer, aggregation-prone Aβ_42_ species by shifting the equilibrium among alternative substrate-binding states, a mechanistic link directly observable within MD-derived and free energy-derived landscapes. Systematic *in silico* screening across comprehensive FAD mutation (Fig. 2) libraries has not only recapitulated experimentally observed shifts in substrate processing ratios but also predicted novel, previously uncharacterized contributors to pathogenicity [41]. From a therapeutic perspective, these mutation-induced conformational shifts have direct implications for Notch-sparing drug design. Computational studies demonstrate that FAD mutations differentially affect APP and Notch engagement by selectively reshaping substrate-binding grooves and allosteric networks [42]. By explicitly modeling mutant-specific energy landscapes, integrative approaches can identify ligand-binding sites and allosteric modulators capable of rebalancing APP cleavage while preserving Notch processing. Such mutation-aware simulations provide a rational framework for designing modulators that restore productive conformational states rather than globally inhibiting γ-secretase activity.

Integrative modeling thus enables a mechanistic “genotype-to-phenotype” pipeline, where computational prediction, biophysical validation, and clinical correlation converge, providing critical insights for precision medicine and mutation-specific modulator development. Importantly, by simulating how specific ligands or allosteric modulators can rebalance these altered energy landscapes, researchers gain predictive capacity for rational, Notch-sparing drug design, which is a fundamental objective in AD therapeutics [31].

## The AI-driven pipeline for next-generation modulator discovery

Numerous small molecules have been identified for Alzheimer’s; however, most of them were rejected due to dose-dependent toxicity identified in the clinical phase. One molecule E2012 has been shown to be a modulator of γ-secretase, binding to its allosteric site of γ-secretase [43]. This review examines how the integration of AI/machine learning (ML) proves useful to identify small molecules as an effective GSM or GSI.

### Machine learning for predicting modulator activity and selectivity

**Figure 3:**
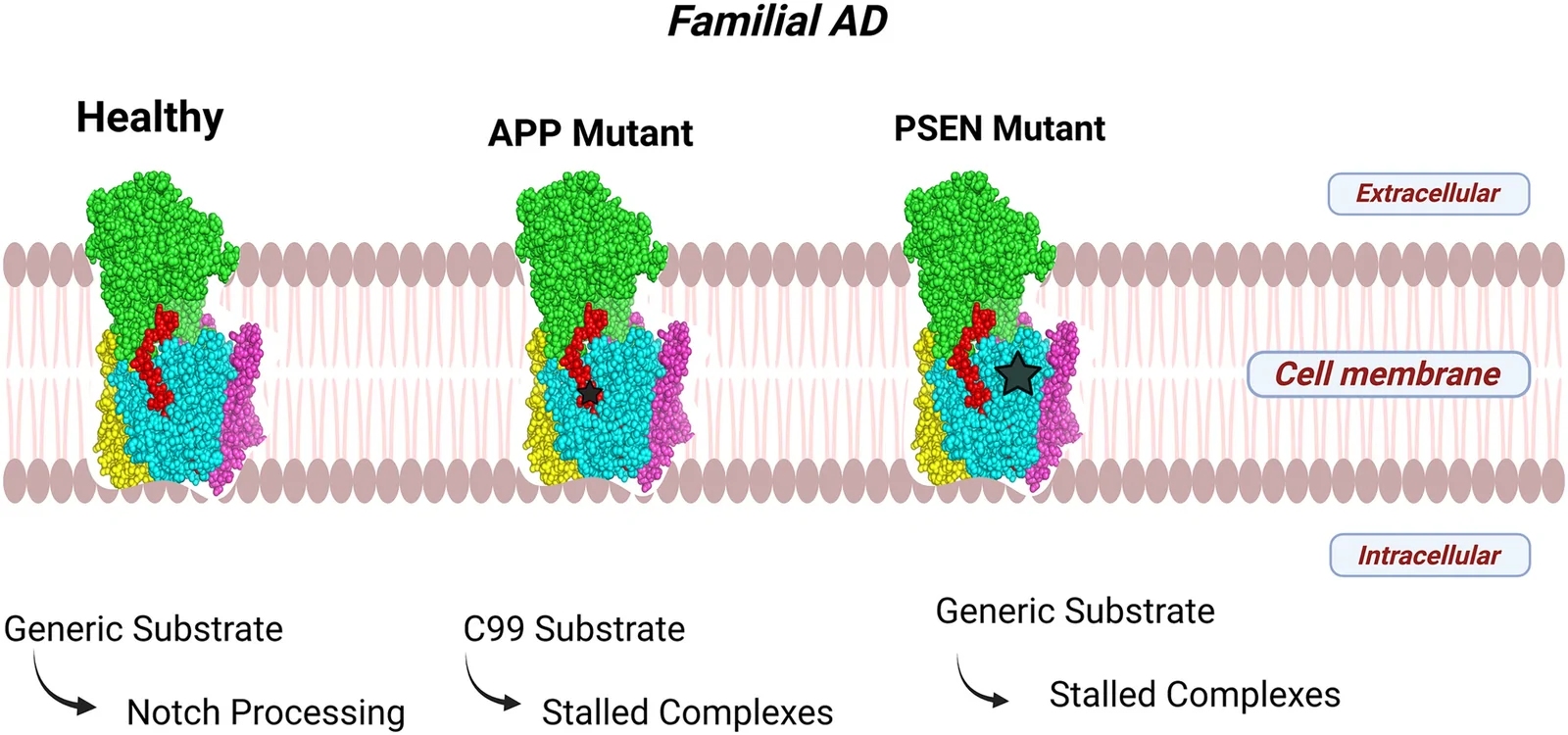
**Molecular consequences of FAD mutations on γ-secretase-substrate processing.** Schematic representation of γ-secretase function under healthy conditions and in the presence of APP or presenilin (PSEN) mutations. In the healthy state, γ-secretase efficiently processes generic substrates, including Notch, resulting in productive cleavage. In contrast, APP and PSEN mutations lead to the formation of stalled γ-secretase–substrate complexes, impairing normal proteolysis and promoting pathogenic outcomes. The star symbols denote stalled or nonproductive enzyme-substrate states, with star size reflecting the relative severity or stability of the stalled complex: larger stars indicate more persistent or energetically stable stalled states, whereas smaller stars represent less severe or transient stalling. The membrane orientation (extracellular, membrane, and intracellular) is indicated for spatial context.

ML has been extensively employed in the identification of the GSM and GSI of AD, significantly reducing the duration of the drug discovery process from 15–5 years. This technology has gained widespread adoption in preclinical studies, particularly in identifying key modulators and inhibitors of γ-secretase (Fig. 3). It enables the prediction of toxicity and Absorption, Distribution, Metabolism, Excretion (ADME) properties as well as the selection of features for ligand binding through quantitative structure–activity relationship (QSAR) models by integrating multimodal datasets [44, 45].

QSAR serves as the initial step for ligand-based drug design, where GSIs or GSMs will be filtered based on their distinct properties, and compounds exhibiting modulatory or inhibitory effects are identified [46].

Recent studies have aimed to develop QSAR models for identifying GSMs and GSIs as potential treatments for AD. A recent study by Sharma *et al*. developed a QSAR model using multiple linear regression, partial least squares, and feed-forward neural network methods to determine various descriptors of 53 GSIs, ensuring compliance with Lipinski’s rule of five [47]. Furthermore, studies have demonstrated that the integration of explainable AI and Read Across Structure Activity Relationships (RASAR) with QSAR simplifies the prediction of their EC50 values [48, 49]. Studies by Ajala *et al*. and Ponzoni *et al*., have developed QSAR models that are effective against AD and identify the inhibitors as good modulators or inhibitors of enzymes [50, 51]. Hence, QSAR can be used as an effective tool to screen potent GSMs and GSIs.

### High-throughput virtual screening of ultra large chemical libraries

The integration of high-resolution cryo-electron microscopy into computational drug discovery has fundamentally reshaped virtual screening. Ensemble docking strategies leverage multiple conformations, such as conformational heterogeneity and dynamic ensembles of macromolecular targets, to model ligand adaptability and conformational selection mechanisms, significantly improving hit identification rates and leading to enrichment.

The ensemble docking methodologies harness multiple conformational states to account for receptor flexibility, thus improving the accuracy and relevance of ligand-binding predictions. Molecular dynamics simulations further validate the ensemble model, indicating that γ-secretase transitions between rigid and flexible conformations are modulated by substrate and lipid interactions [52, 53]. Notably, the binding of GSMs induces conformational shifts that unveil cryptic allosteric sites, as demonstrated through experimental and *in silico* approaches [43, 54, 55]. Numerous studies have used virtual screening for identifying multitarget drugs, commonly known as polypharmacology or multitarget drug design [56]. A study by Oddesson *et al*. employed an HTVS approach to identify modulators of two targets involved in AD: alpha-7 nicotinic acetylcholine receptors and acetylcholine esterase [57].

The integration of AI/ML approaches has substantially accelerated γ-secretase drug discovery. Initial classification efforts employing support vector machines and random forest models have achieved high accuracy in distinguishing γ-secretase inhibitors based on molecular descriptors. Contemporary explainable AI frameworks, such as physicochemical profiling, have decoded substrate recognition logic, facilitating the identification of novel γ-secretase substrates within the human proteome [58]. Graph neural networks (GNNs) and active learning paradigms, exemplified by GraphscoreDTA, GNNSeq, and regression-based iterative screening, have improved protein–ligand binding affinity prediction and reduced the screening library size without compromising hit rates [59–61].

**Figure 4:**
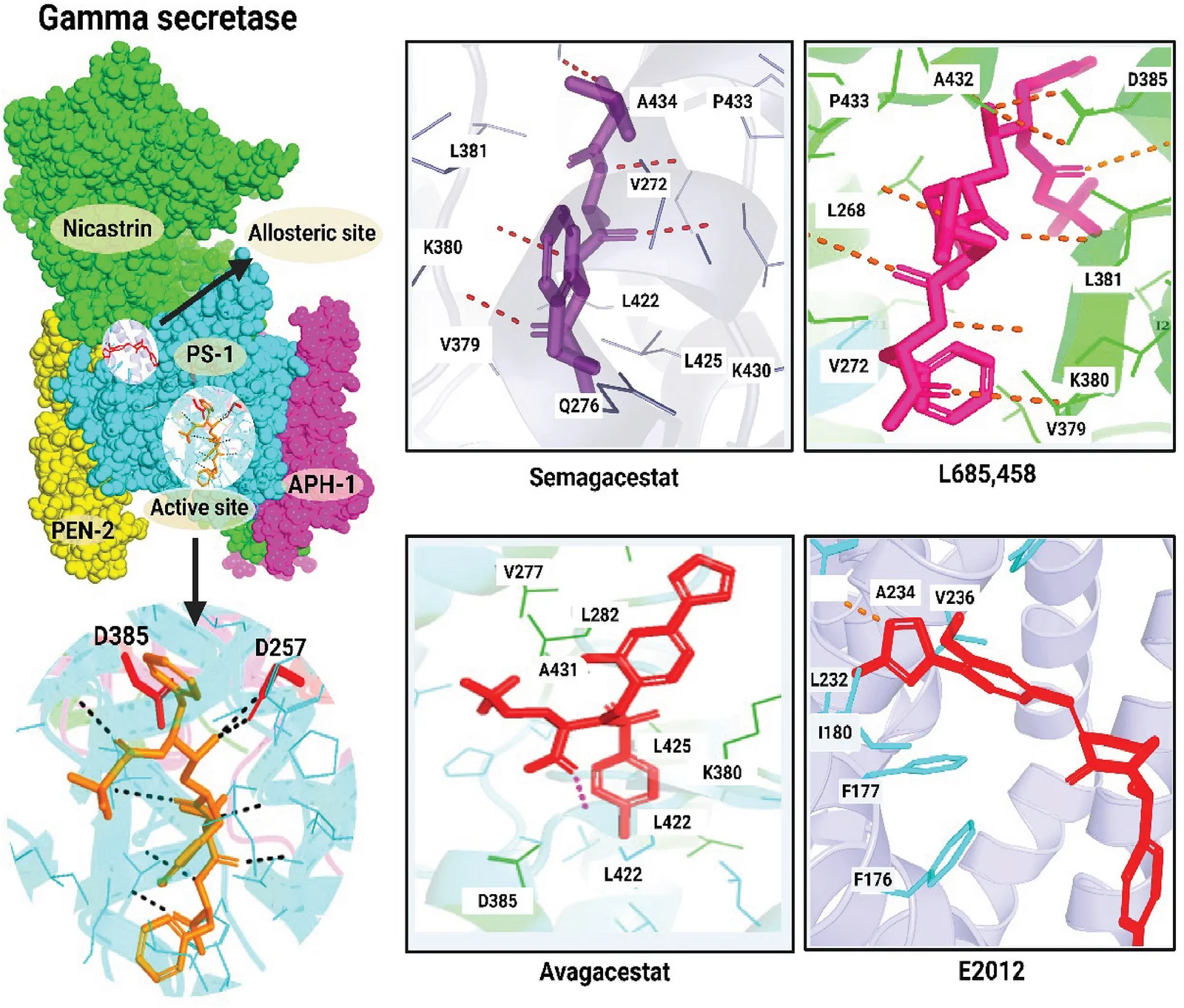
**Schematic molecular basis of γ-secretase processing APP versus Notch and the consequences of inhibition versus modulation (adapted from:** [43]**)**. The panel depicts the multi-subunit γ-secretase complex, highlighting the catalytic active site and allosteric site. High-resolution insects demonstrate the specific binding pockets and molecular interactions of different drug candidates.

Importantly, integrating AlphaFold-predicted structures with cryo-EM-derived ensembles and physics-based docking further expands the repository of addressable conformations for virtual screening (Fig. 4). Cutting-edge protocols such as AlphaRED demonstrate increased success rates for challenging flexible targets by combining data-driven structural templates with conformational sampling and iterative screening [62]. Furthermore, pharmacophore modeling represents a fundamental computational strategy in structure- and ligand-based drug design. This approach abstracts essential molecular features required for optimal protein–ligand recognition into spatially distributed chemical motifs [63]. The pharmacophore concept, defined as an ensemble of steric and electronic features necessary for supramolecular interactions with a specific biological target to trigger or modulate its pharmacological response, has evolved substantially from qualitative feature mapping to quantitative three-dimensional representations incorporating hydrogen bond donors/acceptors, hydrophobic regions, aromatic centers, electrostatic interactions, and metal coordination sites [64]. This approach facilitates rational reduction of the chemical search space in HTVS campaigns, enabling efficient interrogation of vast compound libraries containing millions to billions of molecules while maintaining pharmacologically relevant hit enrichment [65].

GNNs and transformer-based models enable automated extraction of pharmacophoric features from complex biological data while learning nonlinear relationships between molecular descriptors and bioactivity profiles. The pharmacophore-guided molecule generation framework exemplifies this paradigm shift by encoding pharmacophore hypotheses as fully connected graphs via gated graph convolutional networks. In this architecture, nodes represent individual pharmacophoric features while edge attributes capture spatial constraints. Subsequently, transformer-based decoding is employed to generate molecules conforming to specified pharmacophore templates [66]. This architecture achieved validity scores exceeding 98%, uniqueness approaching 100%, and maintained structural novelty while generating compounds exhibiting superior docking affinities compared with ChEMBL-annotated active molecules. Similarly, PharmacoNet employs neural scoring functions to perform ultra large-scale pharmacophore-based screening of hundreds of millions of compounds within hours, demonstrating hit enrichment rates substantially exceeding those of traditional structure-based docking methods for the identification of effective GSMs and GSIs [64, 67].

### Deep generative models for *de novo* modulator design

The advent of generative models has significantly accelerated the drug discovery process, reducing it from years to months and revolutionizing the synthesis of novel medicines. The development of new AI models and the use of existing generative AI models, such as GANs and VAEs, represent transformative approaches in *de novo* modulator design for Alzheimer’s γ-secretase [68, 69].

GANs, a deep learning approach for designing *de novo* molecules, employ two independent adversarial neural networks, the generator and discriminator [69]. Wasserstein GANs (WGAN) integrated with graph convolutional networks, exemplified by the MedGAN, generate scaffold-constrained molecules with 92%–95% novelty while preserving essential drug properties. WGAN implementations using gradient penalties eliminate mode collapse, producing chemically valid, synthesizable modulators for γ-secretase. GAN models such as GraphDRP and DeepChem have shown robust performance in predicting the bioavailability of GSMs and GSIs against γ-secretase [70, 71].

VAEs represent a probabilistic approach to molecular generation through latent space navigation [72]. For the discovery of GSM, VAE-based frameworks such as NP-VAE and Junction Tree-VAE successfully handle complex molecular scaffolds while preserving chirality and generating novel compounds optimized for target functions [73, 74].

Reinforcement learning (RL) is a deep learning technique that trains models using molecular properties as learning objectives, such as graph-based, string, and fingerprint encodings, thus emerging as a transformative approach for *de novo* γ-secretase modulator design, enabling simultaneous optimization of conflicting pharmacological objectives [75, 76]. Furthermore, RL frameworks, such as MolRL and RLDock, have been developed to optimize ligand generation for γ-secretase [77, 78]. The challenge of balancing high potency against Aβ_42_ while maintaining selectivity for APP processing over Notch signaling and preserving drug likeness exemplifies multi-objective optimization in molecular design.

In the realm of γ-secretase modulators, multi-objective RL strategies emerge as a pivotal solution to the critical selectivity challenge where GSI-related Notch inhibition causes clinical trial failure. Contemporary methods such as MOLLM (multi-objective large language model) and ExMolRL leverage in-context learning and Pareto front selection, achieving state-of-the-art performance across multiple objectives [79, 80]. RL-based *de novo* design achieves 65%–95% predicted active compounds for benchmark tasks, generating novel, valid molecules with favorable Absorption, Distribution, Metabolism, Excretion, and Toxicity (ADMET) properties. Integration with ensemble docking across conformational states and AI/ML scoring functions further enhances selectivity optimization, positioning RL as a paradigm-shifting tool for discovering next-generation GSMs with improved therapeutic windows [81].

## Synthesis and future perspectives converging

### Converging computational and experimental validation

**Figure 5:**
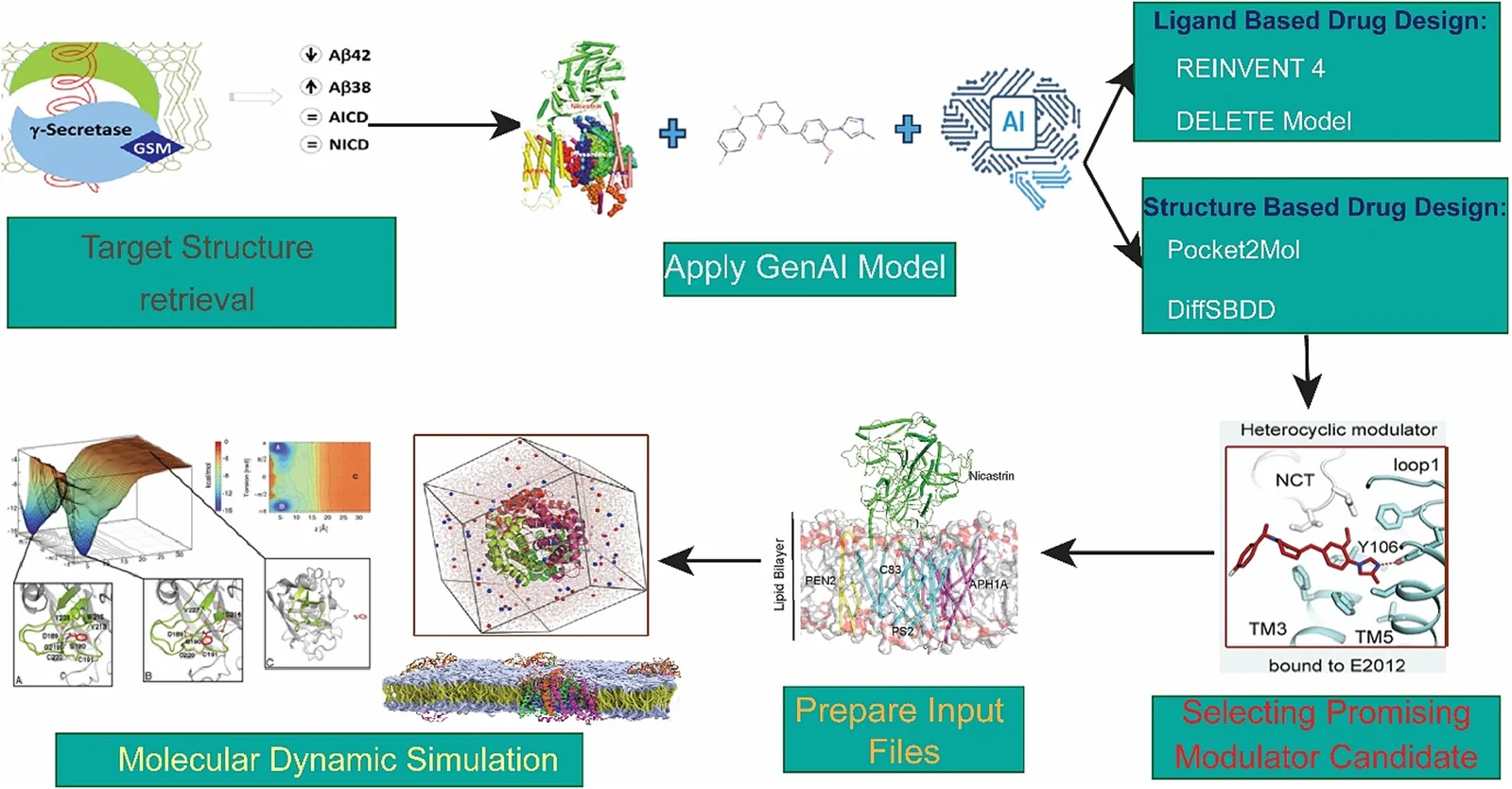
**Integrated computational workflow for the discovery of novel γ-secretase modulators.** This flowchart outlines the strategy of combining generative AI and virtual screening techniques to identify new GSMs. The process involves preparing structural input files, generating novel molecular structures using models like REINVENT 4 and Pocket2Mol, and then screening the candidates against the γ-secretase target. Successful candidates proceed through a selection process and can be used as scaffolds for further optimization, leveraging computational models to analyze their binding and effects on the Aβ_42_/Aβ_38_ ratio.

Understanding exactly where γ-secretase modulators bind and how they work without disrupting Notch signaling has become a major focus of recent computational and experimental studies aimed at developing Alzheimer’s disease-relevant γ-secretase modulators (GSMs) [2]. Recent integrative approaches (Fig. 5) that combine pharmacophore modeling, binding-site screening, molecular docking, and Molecular Mechanics/Generalized Born Surface Area (MMGBSA) free-energy calculations have been used to predict ligand binding affinities and prioritize potential γ-secretase modulator binding pockets within the γ-secretase complex [82]. These analyses uncovered a strong semiquantitative relationship between experimentally measured pIC^50^ values and MMGBSA-derived binding affinities at a specific region of the γ-secretase complex, known as site 4. In contrast, other candidate pockets showed little to no comparable correlation, providing strong support for site 4 as the primary binding site for Notch-sparing γ-secretase modulators. Modulator binding and activity were found to depend on key physicochemical features, such as hydrophobicity, molecular size, and aromatic character, consistent with the largely hydrophobic environment of the transmembrane binding pocket [82]. Taken together, these findings bridge computational predictions with experimental validation to clarify the structural and chemical features that drive selective γ-secretase modulation relevant to Alzheimer’s disease. Binding-site identification was carried out using fragment- and cavity-based mapping tools, including FTMAP and SiteMap, to systematically pinpoint potential ligand-binding regions within the γ-secretase–substrate complex. After the initial mapping step, binding cavities within the transmembrane regions of PS1 were prioritized for further analysis because of their direct involvement in substrate processing and modulation. FTMAP places a library of small fragments onto candidate sites and identifies binding “hotspots” based on fragment clustering, highlighting energetically favorable regions for ligand binding. In parallel, SiteMap detects cavity regions through solvent-accessible surface mapping and assesses their druggability using geometric and physicochemical features. Together, these analyses define both hydrophilic and hydrophobic subregions within each cavity, offering insights into ligand complementarity. Hydrophobic areas are further characterized by hydrogen-bond donor and acceptor properties, enabling the rational design of pharmacophore models [83].

### Remaining challenges and open questions

The γ-secretase complex is a highly dynamic, membrane-embedded protease complex. Its catalytic activity is carried out by presenilin-1 (PS1), while Nicastrin, Aph-1, and Pen-2 contribute to substrate recognition and maintain the stability of the complex in the context of Alzheimer’s disease [84]. This conformational flexibility allows γ-secretase to cleave multiple substrates, most notably APP, whose improper processing produces pathogenic amyloid-β peptides that play a central role in Alzheimer’s disease [85]. Although cryo-EM has revealed several conformational states of γ-secretase, these structures likely represent only a subset of the dynamic ensemble that exists in native neuronal membranes [86]. Within PS1, especially in transmembrane helices TM6 and TM7, local flexibility governs substrate gating, cleavage accuracy, and trimming processivity, directly affecting the Aβ42/Aβ40 ratio in Alzheimer’s disease [87]. A major unresolved challenge is understanding how γ-secretase dynamically shifts between these conformational states during substrate engagement and successive cleavage events. Static structural models cannot fully capture this temporal complexity [88]. Another important open question is the molecular basis of Notch-sparing modulation. Although site 4 has emerged as a key binding pocket for γ-secretase modulators, how ligand binding at this site selectively alters APP processing while sparing Notch signaling remains unclear [82]. Familial Alzheimer’s disease-associated PS1 mutations further complicate modulation strategies, as many of these variants disrupt cleavage precision rather than substrate binding, resulting in increased production of longer, more pathogenic Aβ species [89]. Future advances will depend on ensemble-based, time-resolved approaches that integrate cryo-EM, molecular dynamics simulations, and functional APP cleavage assays to guide the development of safe and effective γ-secretase modulation strategies for AD therapy [2].

## Conclusion

The remarkable convergence of integrative structural biology, atomistic simulations, and AI-driven design has transformed the landscape of membrane protein research for rational drug discovery. The convergence illustrates how computational biology has evolved from static visualization to predictive and hypothesis-generating frameworks, using the γ-secretase complex as an example. These advances provide unparalleled insight into the mechanisms underlying substrate identification, conformational dynamics and the pathogenic implications of familial mutations. AI-driven generative algorithms enable quick discovery and optimization of selective γ-secretase modulators without affecting crucial Notch signaling. These techniques are promising for developing safer, more effective treatments for Alzheimer’s disease and related illnesses. Despite major progress, several challenges remain. Capturing the full range of the dynamic states of γ-secretase and understanding the fine details of its allosteric regulation remains a formidable task. To address these issues, continuous feedback between computational predictions and experimental validation will be crucial for refining mechanistic models to improve their accuracy. Through the synergistic use of cryo-EM models, enhanced sampling techniques, and AI-driven virtual screening, unprecedented insights have emerged into substrate specificity, dynamic conformational landscapes, and the impact of disease-associated mutations. This revealed the intricacies of substrate docking, active site accessibility, and allosteric modulation. This review provides a framework for predictive drug discovery that leads to safer, more selective therapies for γ-secretase and other intramembrane proteases. Ongoing integration of computational and experimental workflows will be important for validating models, improving mechanistic hypotheses and generating translational impact in neurodegeneration diseases. Future research should focus on understanding the dynamic heterogeneity of γ-secretase and allosteric regulation to develop targeted therapeutics. A computational understanding of γ-secretase is crucial for overcoming the Notch-sparing modulation challenge, enabling the identification and optimization of compounds that fine-tune amyloid-β production while maintaining the integrity of critical signaling pathways such as the Notch pathway. In conclusion, the trajectory of γ-secretase research highlights the revolutionary role of computational biology, which transcends mere description to encompass prediction and design. This approach provides a clear, mechanistically grounded path to safer, more effective and disease-specific medicines. These tools allow researchers to visualize the assembly of the enzyme’s four subunits, the selective recruitment of substrates such as APP or Notch, and the subtle structural shifts caused by FAD mutations that alter their activity. Using enhanced molecular dynamics methods, including umbrella sampling, steered MD, and replica exchange, scientists can simulate complex molecular movements and energetic pathways, revealing how γ-secretase processes different substrates and identifying potential points where selectivity can be engineered. One of the most significant challenges in drug discovery for AD has been the Notch-sparing dilemma: traditional inhibitors of γ-secretase interfere not only with pathogenic amyloid beta production but also with essential Notch signaling, resulting in undesirable side effects. The integration of computational predictions with experimental feedback has already led to discoveries validated both *in silico* and at the bench, propelling the field forward. Moreover, this approach provides a versatile blueprint extending beyond AD by offering strategies applicable to other membrane proteases and conditions requiring precise functional control. In summary, computational biology is not just improving how membrane protein systems, such as γ-secretase are studied; it fundamentally alters the boundaries of what is achievable. This transformation not only accelerates drug discovery for AD but also offers a roadmap for designing precise, mechanism-based treatments for other difficult targets in neurodegeneration, cancer, and therapeutic discovery.

## Future prospective

The integration of computer-based and experimental methods is used to determine how we understand and develop treatments for γ-secretase and other complex membrane proteins. A robust feedback system wherein computer models guide mutagenesis, laboratory experiments, and structural studies and experimental outcomes are subsequently utilized to modify those models would help rapid translation of theoretical findings into tangible medical applications. Recent advancements in γ-secretase research have demonstrated the potency of this approach. Predictions established through computational methods have been corroborated in the laboratory, demonstrating the strength and reliability of computer-aided drug discovery. However, many challenges remain. One major difficulty lies in the flexible and dynamic nature of the γ-secretase complex. To comprehensively capture all its functional conformations and transient states, we require advanced tools for both simulation and experimentation. Another key goal is to understand the precise mechanism by which γ-secretase modulators bind to the protein and induce selective allosteric effects, which is essential for designing more selective and effective drugs. Predicting the side effects and undesirable interactions of drugs targeting complex membrane proteins is also a major concern, highlighting the need for better computational models and stronger links between chemistry, biology, and genomics. The computational approaches built for γ-secretase can serve as valuable models for studying other similar enzymes, such as signal peptide peptidases (SPPs), and for exploring membrane-related pathways implicated in cancer and many other diseases beyond AD. These integrated methodologies will help scientists understand molecular mechanisms, anticipate drug behavior, and develop tailored treatments for a wide range of medical issues. As computing power continues to expand and experimental methods improve, we are poised to routinely design medications for “undruggable” membrane proteins. This advancement will significantly broaden the application of precision medicine in the treatment of neurodegenerative illnesses, cancer, and a multitude of other conditions.

**Key Points**

- Cryo-EM and integrative modeling reveal γ-secretase structure and substrate recognition.
- Molecular dynamics expose catalytic motions, selectivity, and mutation effects.
- AI/ML and generative models accelerate discovery of selective γ-secretase modulators.
- Computational and experimental synergy enables Notch-sparing modulator design.
- A generalizable blueprint for intramembrane proteases combines structural, dynamic, and AI-driven strategies for γ-secretase, providing a transferable framework for studying neurodegeneration.

## Data Availability

No new data was created or analyzed in this study. Data sharing is not applicable to this article.
